# Incidence and risk factors for bone metastases at presentation in solid tumors

**DOI:** 10.3389/fonc.2024.1392667

**Published:** 2024-05-10

**Authors:** Brendan J. Knapp, Giordano F. Cittolin-Santos, Mary E. Flanagan, Nikhil Grandhi, Feng Gao, Pamela P. Samson, Ramaswamy Govindan, Daniel Morgensztern

**Affiliations:** ^1^ Department of Medicine, Division of Oncology, Washington University School of Medicine in St. Louis, St. Louis, MO, United States; ^2^ Department of Surgery, Division of Public Health Sciences, Washington University School of Medicine in St. Louis, St. Louis, MO, United States; ^3^ Department of Radiation Oncology, Washington University School of Medicine in St. Louis, St. Louis, MO, United States

**Keywords:** bone metastases, risk factors, lung cancer, breast cancer, prostate cancer

## Abstract

**Introduction:**

Bone metastases are associated with increased morbidity and decreased quality of life in patients with solid tumors. Identifying patients at increased risk of bone metastases at diagnosis could lead to earlier interventions. We sought to retrospectively identify the incidence and predictive factors for bone metastases at initial diagnosis in a large population-based dataset.

**Methods:**

The Surveillance, Epidemiology, and End Results (SEER) database was used to identify patients 18 years-old or older diagnosed with solid cancers from 2010 to 2019. Patients with hematologic malignancies and primary tumors of the bone were excluded. We calculated the incidence and predictive factors for bone metastases according to demographic and tumor characteristics.

**Results:**

Among 1,132,154 patients identified, 1,075,070 (95.0%) had known bone metastasis status and were eligible for the study. Bone metastases were detected in 55,903 patients (5.2% of those with known bone metastases status). Among patients with bone metastases, the most common primary tumors arose from lung (44.4%), prostate (19.3%), breast (12.3%), kidney (4.0%), and colon (2.2%). Bone metastases at presentation were most common in small cell lung cancer (25.2%), non-small cell lung cancer (18.0%), and esophageal adenocarcinoma (9.4%). In addition to stage classification, predictors for bone metastases included Gleason score (OR 95.7 (95% CI 73.1 – 125.4) for Grade Group 5 vs 1 and OR 42.6 (95% CI 32.3 – 55.9) for Group 4 vs 1) and PSA (OR 14.2 (95% CI 12.6 – 16.0) for PSA > 97 vs 0 – 9.9) for prostate cancer, HER2 and hormonal receptor (HR) status (OR 2.2 (95% CI 1.9 – 2.6) for HR+/HER2+ vs HR-/HER2-) for breast cancer, histology (OR 2.5 (95% CI 2.3 – 2.6) for adenocarcinoma vs squamous) for lung cancer, and rectal primary (OR 1.2 (95% 1.1 – 1.4) vs colon primary) and liver metastases (OR 8.6 (95% CI 7.3 – 10.0) vs no liver metastases) for colorectal tumors.

**Conclusions:**

Bone metastases at presentation are commonly seen in solid tumors, particularly lung, prostate, breast, and kidney cancers. Clinical and pathologic factors are associated with a significantly increased risk for bone metastases.

## 1 Introduction

Bone metastases are frequently detected in patients with advanced solid tumors, most commonly involving the axial skeleton, including spine, pelvis, ribs, skull, proximal humeri and femora, reflecting the distribution of red bone marrow where the highly vascular tissue provides a microenvironment that promotes cellular growth (1). Patients with bone metastases are usually incurable and often symptomatic with pain and other complications known as skeletal related events (SRE), including pathological fractures, need for radiation therapy to improve the bone pain, surgery to prevent or repair a fracture, spinal cord compression, and hypercalcemia. The SREs are usually associated with loss of mobility and diminished social function, reduced quality of life, worse survival, and increased medical costs (2). The most common SRE is severe bone pain, which occurs in up to 80% of patients with bone metastases (3). The diagnosis of bone metastases earlier in the course of disease may allow pre-emptive intervention, limiting SREs, preserving function and quality of life.

In a retrospective review using the Oncology Services Comprehensive Electronic Records (OSCER) of patients diagnosed between 2004 and 2013 including 382,733 patients aged 18 or older, the median time to bone metastases from diagnosis was 69 days, with 2.9% of solid cancer patients diagnosed with bone metastases at 30 days, 4.8% at 1 year, 5.6% at 2 years, 6.9% at 5 years and 8.4% at 10 years (4). In a retrospective analysis of the Surveillance, Epidemiology, and End Results (SEER) database from 2010 to 2015, 5.1% of patients had bone metastases at diagnosis (5). However, neither study reported clinicopathologic risk factors associated with the diagnosis of bone metastases, other than stage and histology.

Identifying specific features that are predictive of bone metastases at initial diagnosis could allow dedicated screening and potentially earlier intervention. Since there are limited data on the risk factors for bone metastases at presentation in patients with solid tumors, we performed a retrospective analysis of the SEER database evaluating the clinical predictors including demographics and tumor characteristics, using the tumor markers that were not previously available.

## 2 Materials and methods

The SEER database [SEER Datasets and Software (RRID: SCR_003293)], a registry program covering approximately 48% of the US population, was used to identify patients 18 years-old or older with solid tumors diagnosed between 2010 and 2019. Patients with lymphoma, other hematologic malignancies, primary tumors of the bone, and those diagnosed by autopsy or death certificate were excluded. Patients with unknown bone metastasis status were also excluded. Tumors were classified according to the International Classification of Diseases (3^rd^ Edition) codes listed for histology in the database and, the staging was based on the American Joint Committee on Cancer, either the 7^th^ edition for patients diagnosed before 2018 or the 8^th^ edition for those diagnosed in 2018 or 2019 (6, 7).

A broad classification of primary tumors was used to calculate their percentages among patients with bone metastases at presentation, whereas the percentages of bone metastases according to the primary tumors was calculated for the most common histologies to decrease heterogeneity. The incidence and predictive factors for bone metastases at the initial diagnosis of malignancy were calculated according to demographics and baseline tumor characteristics. Prostate-specific antigen (PSA) was the only serologic factor used in the study and was classified according to the SEER data into <10, 10 to 19, 20-97 and ≥ 98 ng/mL.

Data analyses were descriptive in nature and performed separately in the primary tumor cohorts with the highest incidence of bone metastases. In each cohort, the patient demographic and clinical characteristics were summarized using means and standard deviations or counts and frequencies as appropriate. Univariate and multivariate logistic regression models were used to assess the association between patient characteristics and bone metastasis, with the strength of association described by odds ratios (OR) of bone metastases. Variables included sex, race, tumor characteristics such as histology and stage, and biomarkers when pertinent. Age was specifically included in analysis of breast cancer patients due to its importance in evaluating menopausal status. For patients with colorectal cancer, unlike the other major cancers evaluated, the liver is by far the most common site of initial metastases. Therefore, the presence of liver metastases becomes an important variable for colorectal cancer, particularly when evaluating the probability of bone metastases without liver involvement. All the multivariate models were performed as complete case analysis where all cases with missing data on any variables are removed from the analysis. The variables in each multivariate model were pre-selected based on clinical considerations rather than statistical significance. For the cohort with colorectal adenocarcinoma, because missing values in tumor stage and nodal stage led to substantial loss of cases with bone metastasis, these 2 variables were not included in the multivariate model. Data analyses were performed using the statistical package SAS version 9.4 (SAS institute, Cary, NC). All p-values were two-tailed, and the probability of type I error was set at 0.05.

## 3 Results

### 3.1 Most common tumors with bone metastases at presentation

**Figure 1:**
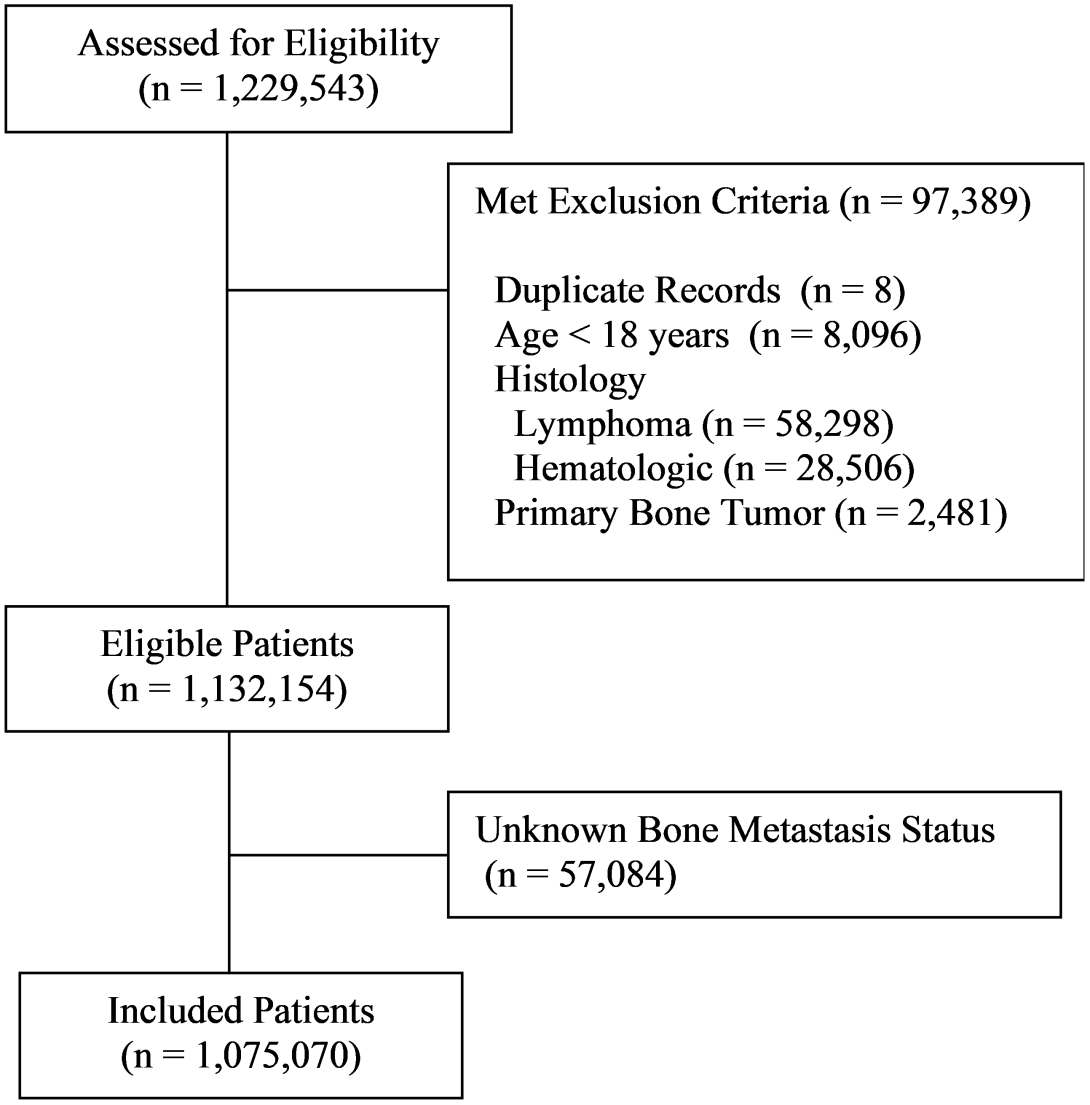
Flowchart of patients meeting inclusion criteria.

Among the 1,229,543 patient records included in the database, 97,389 were excluded due to not meeting eligibility criteria, of which there were eight duplicate records, 8,096 patients less than 18 years of age, 58,298 with lymphoma, 28,506 with other hematologic malignancies, and 2,481 with primary bone tumors (**Figure 1**). Among the remaining 1,132,154 patients, 1,075,070 (95.0%) had known bone metastasis status and were included in the study. Bone metastases were detected in 55,903 patients representing 4.9% of all eligible patients and 5.2% of those with known bone metastasis status. Patient demographics are provided in **Supplementary Table 1**.

**Figure 2:**
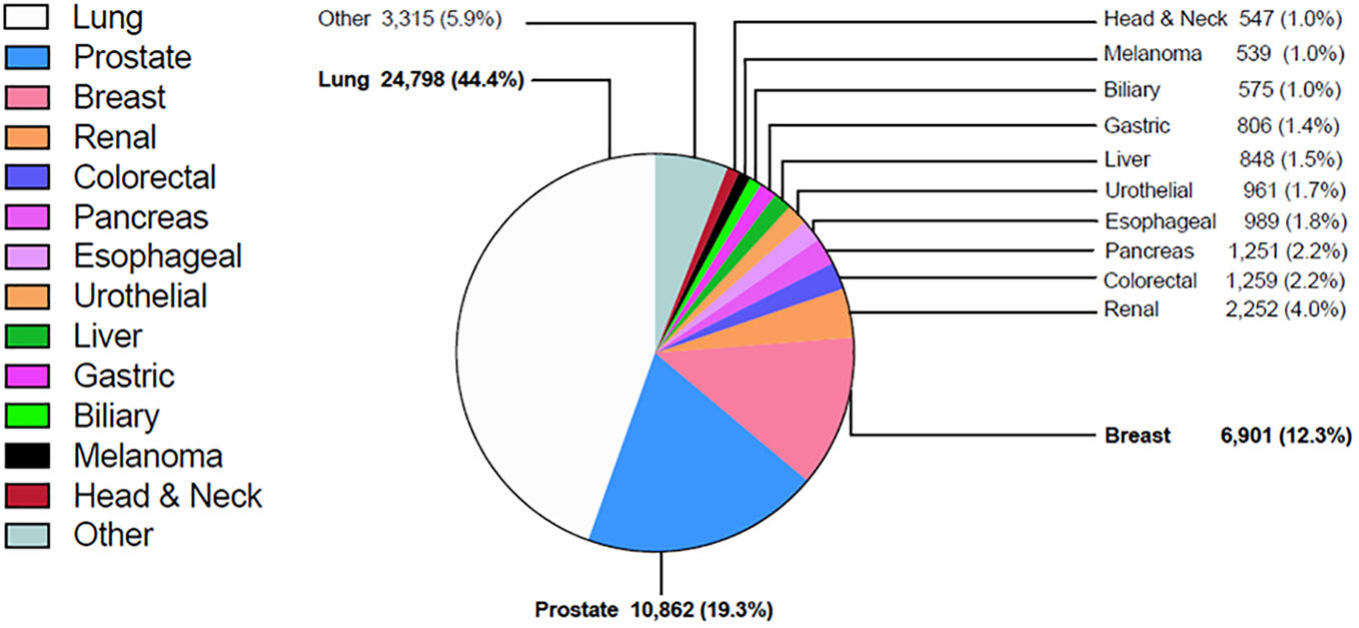
Distribution of primary tumor location in patients with bone metastases.

Among patients with bone metastases, the most common primary tumors were lung (44.4%), prostate (19.3%), breast (12.3%), kidney (4.0%), colorectal (2.2%) and pancreas (2.2%) (**Figure 2**). Among the more common tumors, the highest percentage of bone metastases at diagnosis were small cell lung cancer (25.2%), non-small cell lung cancer (NSCLC) (18.0%), and esophageal adenocarcinoma (9.4%) (**Table 1**).

**Table 1:** Incidence of bone metastases at presentation according to the primary tumor.

Tumor Location and Histology	Patients with Bone Metastases	Total Patients	Percentage with Bone Metastases
Lung	24,798	133,396	18.59
**Non-Small Cell Lung Cancer**	18,123	100,448	18.04
*Squamous*	2,908	26,539	10.96
*Adenocarcinoma*	12,930	63,257	20.44
*Large Cell*	300	1,587	18.90
*Other*	1,985	9,065	21.90
**Small Cell**	3,814	15,159	25.16
**Carcinoid/Neuroendocrine**	419	4,318	9.70
**Other**	2,442	13,471	18.13
Breast	6,901	198,025	3.48
*Infiltrating Ductal*	4,490	154,626	2.90
*Inflammatory*	69	332	20.78
*Lobular*	1,209	30,995	3.90
*Other*	1,113	12,072	9.22
Prostate	10,862	15,7261	6.91
*Adenocarcinoma*	9,071	153,325	5.92
*Small cell*	86	191	45.03
*Other*	1,705	3,745	45.53
Gastrointestinal			
**Esophageal**	989	11,644	8.49
*Adenocarcinoma*	712	7,547	9.43
*Squamous*	200	3,450	5.80
*Other*	77	647	11.90
**Gastric**	806	17,629	4.57
*Adenocarcinoma*	730	14,054	5.19
*Other*	76	3,575	2.13
**Colorectal**	1,259	97,860	1.29
*Adenocarcinoma*	1,043	89,510	1.17
*Neuroendocrine*	52	4,516	1.15
*Squamous*	10	604	1.66
*Other*	154	3,230	4.77
**Small bowel**	97	6,711	1.45
**Appendix**	21	3,795	0.55
**Anal**	57	5,454	1.05
**Other gastrointestinal**	278	2,928	9.49
**Liver**	848	19,987	4.24
*Hepatocellular Carcinoma*	756	18,613	4.06
*Other*	92	1,374	6.70
**Pancreas**	1,251	33,252	3.76
*Adenocarcinoma*	896	22,020	4.07
*Ductal Carcinoma*	30	3,726	0.81
*Neuroendocrine*	108	3,019	3.58
*Other*	217	4,487	4.84
**Biliary**	575	11,428	5.03
*Cholangiocarcinoma*	324	4,911	6.60
*Adenocarcinoma*	207	5,431	3.81
*Other*	44	1,086	4.05
Genitourinary			
**Renal**	2,252	42,976	5.24
*Clear cell*	1,774	31,551	5.62
*Transitional*	149	1,495	9.97
*Other*	329	9,930	3.31
**Urothelial**	961	56,924	1.69
*Transitional*	708	53,846	1.31
*Other*	253	3,078	8.22
Head and Neck	547	41,718	1.31
*Squamous*	329	35,536	0.93
*Other*	218	6,182	3.53
Melanoma	539	71,556	0.75
Thyroid	281	38,186	0.74
Endometrial	378	42,772	0.88
Ovarian	205	18,535	1.11
Other	1,998	63,033	3.1700

### 3.2 Non-small cell lung cancer

For patients with NSCLC, bone metastases at presentation were more common in adenocarcinomas (20.4%) than squamous cell carcinomas (11.0%) (p < 0.001). The percentage of bone metastasis increased from 11.4% in stages T0-T2 to 24.9% in stages T3-4, and from 7.6% in N0 to 18.1% in N1 and 28.3% in N2-N3 (p < 0.001)(**Table 2**). In multivariable analysis, the strongest predictors for bone metastases were N2-N3 (OR 4.3 compared to N0 (95% confidence interval [CI] 4.1 – 4.5), adenocarcinoma histology (OR 2.5 compared to squamous (95% CI 2.3 – 2.6)), and N1 (OR 2.5 compared to N0 (95% CI 2.4 – 2.7)).

**Table 2:** Predictors for bone metastases at presentation in non-small cell lung cancer.

Covariate	Group	Total Patients (N = 100,448)	Bone Metastases (N=18,123) (%)	Parametric P-value	Multivariable Analysis OR (95% CI)
**Sex**	Female	48,637	8,028 (16.51)	<0.001	Male vs Female: 1.23 (1.19 – 1.28)
	Male	51,811	10,095 (19.48)		
**Race**	White	79,709	13,973 (17.53)	<0.001	
	Black	9,141	1,600 (17.5)		Black vs White: 0.91 (0.85 – 0.97)
	Other	11,361	2,518 (22.16)		Other vs White: 1.20 (1.13 – 1.26)
**Tumor Subtype**	Squamous Cell	26,539	2,908 (10.96)	<0.001	
	Adenocarcinoma	63,257	12,930 (20.44)		Adenocarcinoma vs SCC: 2.46 (2.34 – 2.58)
	Large Cell	1,587	300 (18.9)		Large cell vs SCC: 1.83 (1.58 – 2.13)
	Other	9,065	1,985 (21.9)		Other vs SCC: 2.16 (2.01 – 2.33)
**Tumor Stage**	T0/1/2	54,141	6,180 (11.41)	<0.001	
	T3/4	36,182	9,025 (24.94)		T3/4 vs T0/1/2: 1.89 (1.82 – 1.96)
**Nodal Stage**	N0	45,658	3,454 (7.56)	<0.001	
	N1	8,198	1,480 (18.05)		N1 vs N0: 2.54 (2.37 – 2.73)
	N2/3	40,814	11,554 (28.31)		N2/3 vs N0: 4.31 (4.12 – 4.51)

### 3.3 Prostate adenocarcinoma

**Figure 3:**
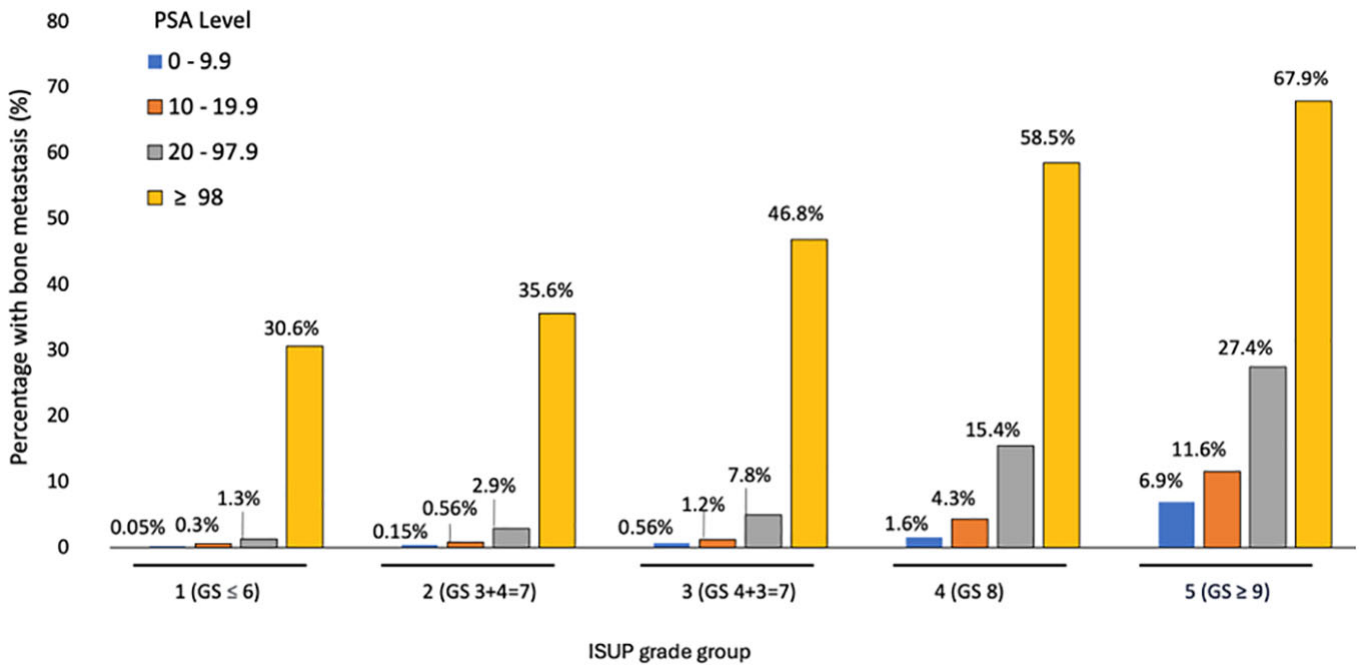
Bone metastases according to ISUP Gleason score group and PSA.

In prostate cancer, the percentage of bone metastases at presentation was higher in T3-4 than T0-2 (10.1% vs 3.4%) and N1 compared to N0 (38.1% vs 3.3%) (p < 0.001), increased from 0.2% in patients with International Society of Urological Pathology (ISUP) Gleason score group 1 to 22.8% in group 5 and from 0.7% in patients with PSA < 10 to 19.5% in those with PSA ≥ 98 ng/dL (p < 0.001). (**Table 3**). In multivariable analysis, the strongest predictors for bone metastases were Gleason ISUP group 5 (OR 95.7 compared to group 1 (95% CI 73.1 – 125.4)), Gleason ISUP group 4 (OR 42.5 compared to group 1 (95% CI 32.3-55.6)) and Gleason ISUP group 3 (OR 16.1 compared to group 1 (95% CI 12.1 – 21.4)). The percentage of bone metastases ranged from 0.05% in patients with Gleason ISUP group 1 and PSA less than 10 to 67.9% in those with ISUP 5 and PSA ≥ 98 (**Figure 3**).

**Table 3:** Predictors for bone metastases at presentation in prostate adenocarcinoma.

Covariate	Group	Total Patients (N = 153,325)	Bone Metastases (N=9,071)(%)	Parametric P-value	Multivariable Analysis OR (95% CI)
**Race**	White	119,733	6,936 (5.79)	<0.001	
	Black	19,257	1,247 (6.48)		Black vs White: 0.94 (0.84 – 1.05)
	Other	11,171	840 (7.52)		Other vs White: 1.08 (0.95 – 1.23)
**ISUP Gleason Group**	1	53,171	84 (0.16)	<0.001	
	2	40,026	241 (0.6)		ISUP 2 vs 1: 4.37 (3.22 – 5.94)
	3	20,565	524 (2.55)		ISUP 3 vs 1: 16.12 (12.12 – 21.44)
	4	16,047	1,382 (8.61)		ISUP 4 vs 1: 42.53 (32.34 – 55.92)
	5	16,588	3,786 (22.82)		ISUP 5 vs 1: 95.73 (73.09 – 125.37)
**Tumor Stage**	T0/1/2	99,084	3,321 (3.35)	<0.001	
	T3/4	15,674	1,579 (10.07)		T3/4 vs T0/1/2: 0.80 (0.73-0.87)
**Nodal Stage**	N0	138,351	4,532 (3.28)	<0.001	
	N1	7,113	2,707 (38.06)		N1 vs N0: 3.97 (3.60 – 4.37)
**PSA Category**	0-9.9	70,683	525 (0.74)	<0.001	
	10-19	17,823	585 (3.28)		PSA 10 – 19 vs 0 – 9.9: 2.28 (1.97 – 2.62)
	20-97	10,071	1,697 (16.85)		PSA 20 – 97 vs 0 – 9.9: 6.76 (5.97 – 7.66)
	>=98	19,943	3,887 (19.49)		PSA >97 vs 0 – 9.9: 14.16 (12.57 – 15.95)

### 3.4 Breast cancer

Among patients with breast cancer, the percentage of bone metastases was higher in inflammatory (20.8%) than lobular (3.9%) or infiltrating ductal (2.9%) carcinomas.

In infiltrating ductal carcinoma, bone metastases were more common in patients with human epidermal growth factor 2 (HER2) positive tumors (4.9% vs 2.7%), higher tumor grade (4.1% for grade III, 3.1% for grade II and 1.0% for grade I), tumor stage T3-4 compared to stage T0-2 (15.8% vs 1.4%) and N2-3 (10.6%) compared to N1 (6.0%) and N0 (0.9%) (all p < 0.001). On multivariable analysis, the strongest predictors for bone metastases at presentation included T3-4 [OR 6.7 compared to T0-2 (95% CI 6.2 – 7.3)], N2-3 (OR 6.1 compared to N0 (95% CI 5.4 – 6.8)) and N1 (OR 4.9 compared to N0 [95% CI 4.4 – 5.3)] (**Table 4**).

**Table 4:** Predictors for bone metastases at presentation in infiltrating ductal carcinoma of the breast.

Covariate	Group	Total Patients (N =54,626)	Bone Metastases (N=4,490) (%)	Parametric P-value	Multivariable Analysis OR (95% CI)	
**Age**	Age<50 years	32,060	1,013 (3.16)	0.002		
	50-59 years	36,275	1,088 (3)		50 – 59 vs < 50 yrs: 1.21(1.09 – 1.33)	
	60-69 years	42,581	1,162 (2.73)		60 – 69 vs < 50 yrs: 1.27(1.15 – 1.40)	
	Age≥70 years	43,710	1,227 (2.81)		>70 vs < 50 yrs: 1.30(1.18 – 1.44)	
**Race**	White	118,643	3,298 (2.78)	<0.001		
	Black	14,253	630 (4.42)		Black vs White: 1.23(1.11 – 1.37)	
	Other	20,820	548 (2.63)		Other vs White: 0.88 (0.80 – 0.98)	
**Hormone Status**	HR-/HER-	16,534	365 (2.21)	<0.001		
	HR+/HER2+	16,723	759 (4.54)		HR+/HER2+ vs HR-/HER2-: 2.22 (1.93 – 2.56)	
	HR+/HER2-	107,384	2,778 (2.59)		HR+/HER2- vs HR-/HER2-: 1.79 (1.58 – 2.02)	
	HR-/HER2+	7,042	284 (4.03)		HR-/HER2+ vs HR-/HER2-: 1.51 (1.26 – 1.80)	
**Tumor Stage**	T0/1/2	138,418	1,936 (1.4)	<0.001		
	T3/4	12,791	2,019 (15.78)		T3/4 vs T0/1/2: 6.70 (6.20 – 7.25)	
**Nodal Stage**	N0	108,144	959 (0.89)	<0.001		
	N1	34,419	2,054 (5.97)		N1 vs N0: 4.86 (4.44 – 5.32)	
	N2/3	10,170	1,073 (10.55)		N2/N3 vs N0: 6.06 (5.44 – 6.75)	

### 3.5 Colorectal adenocarcinoma

In colorectal adenocarcinoma, bone metastases were more common in rectal tumors compared to colon (1.4% vs 1.1%), T3-4 compared to T0-2 (0.8% vs 0.5%) and N2-N3 compared to N0-N1 (1.2% vs 0.8%) (all p < 0.001). Bone metastases were more common in patients with liver metastases (5.7% vs 0.4%) and lung metastases (9.9% vs 0.7%) (p < 0.001). On multivariable analysis, the strongest predictors for bone metastases included liver metastases (OR 8.6 compared to no liver metastases [95% CI 4.3 – 10.0)] and lung metastases (OR 4.7 compared to no lung metastases [95% CI 4.7 – 5.4)] (**Table 5**). T and N staging were not included in the multivariable analysis because values were missing in 56% of patients with bone metastases, which is not unexpected in patients with stage IV colorectal at presentation.

**Table 5:** Predictors for bone metastases at presentation in colorectal adenocarcinoma.

Covariate	Group	Total Patients (N = 89,510)	Bone Metastases (N=1,043) (%)	Parametric P-value	Multivariable Analysis OR (95% CI)
**Sex**	Female	41,871	413 (0.99)	<0.001	
	Male	47,639	630 (1.32)		Male vs Female: 1.18 (1.04 – 1.35)
**Race**	White	68,944	755 (1.1)	<0.001	
	Black	8,308	138 (1.66)		Black vs White: 1.26 (1.04 – 1.53)
	Other	11,720	147 (1.25)		Other vs White: 1.13 (0.93 – 1.36)
**Tumor Location**	Colon	63,560	668 (1.05)	<0.001	
	Rectum	25,541	366 (1.43)		Rectum vs Colon: 1.24 (1.08 – 1.42)
**Liver Metastases**	No	76,656	302 (0.39)	<0.001	
	Yes	12,717	729 (5.73)		Liver Mets vs No Liver Mets: 8.58 (7.34 – 10.03)
**Lung Metastases**	No	84,692	568 (0.67)	<0.001	
	Yes	4,465	441 (9.88)		Lung Mets vs No Lung Mets: 4.66 (4.02 – 5.40)

## 4 Discussion

Our study showed that bone metastases are detected at diagnosis in 5.2% of adult patients with solid tumors. Among patients with bone metastases at presentation, the most primary tumors were lung, prostate, and breast cancers, likely reflecting both their overall incidence and propensity to metastasize to the bones. Furthermore, histology, tumor and nodal stage were predictive for bone metastases.

In patients with NSCLC, adenocarcinoma histology, tumor and nodal stage were independent predictors for bone metastases at presentation, with N2 or N3 adenocarcinoma representing the strongest predictors in multivariable analysis. An earlier study using the SEER database to develop a machine learning algorithm in order to create a web-based predictor for bone metastases in NSCLC (8). Despite using the same database, multiple patients were excluded due to unknown histologic grade and marital status, decreasing the study population from 158,221 to 50,581 patients and likely introducing some degree of bias in the results, particularly with smaller tissue obtained in those with metastatic disease not allowing a full description of the tumor grade. This may explain the higher incidence of bone metastases in our study. Furthermore, since the bone metastases reported at the SEER database are from baseline characteristics, it is unclear why chemotherapy was used in the multivariable analysis. In a large Danish population-based cohort study involving 29,720 patients diagnosed between 1999 and 2010, 340 patients (1.1%) had bone metastases at diagnosis, of which 86 (0.3% of total patients) had also SREs (9). Among the 254 patients (0.9%) with bone metastases at diagnosis without SREs, 155 (61%) subsequently developed SREs. Among the 29,380 patients (98.9%) without bone metastases at diagnosis, 1,692 (5.8%) developed during follow-up, including 905 (53.5%) with SREs. Overall, the cumulative risk of developing bone metastases for all histologies in 1 and 3 years was 5.9% and 6.7% respectively with corresponding cumulative risk for SREs of 55% and 56.7% respectively among those with bone metastases.

In prostate adenocarcinoma, the strongest predictors for bone metastases at presentation in our study were PSA and Gleason ISUP group. In a retrospective study involving 683 patients with prostate cancer diagnosed between 1990 and 1993, PSA levels were associated with the presence of bone metastases in newly diagnosed prostate cancer evaluated with bone scan (10). Bone metastases ranged from 0% in those with levels below 10 to 40% in those with levels above 50. The authors recommended bone scans only for patients with stage T3 or above or poorly differentiated disease. In a review of 23 studies including 8,644 patients who underwent bone scan for newly diagnosed prostate cancer, 1,453 (16.8%) had positive scans (11). Bone metastases were detected in 2.3% of those with PSA less than 10, 5.3% of those with PSA between 10 and 19.9, 16.2% of those with PSA 20 to 49.9, 39.2% of those with PSA 50 to 99.9 and 73.4% of those with PSA 100 or greater. Bone metastases were more common in patients with Gleason score 8 or greater than up to 7 (29.9% versus 5.6%) and in patients with T3 to T4 tumors compared to T1 to T2 (49.5% versus 6.4%). One of the main differences between our study and the retrospective review was the larger number of patients in the SEER database, which allowed a more detailed subdivision of the Gleason and PSA scores as well as a better evaluation of the contribution from each factor. A separate study using the SEER database also found tumor and nodal stage, Gleason score, and PSA as predictive factors for diagnosis of bone metastases in prostate cancer patients (12). Despite the large number of patients included in the study, the population was more heterogeneous due to the presence of other subtypes of prostate cancer in addition to adenocarcinoma, 10,120 patients were excluded due to the lack of known histology grade despite its limited value compared to Gleason score, and 30,989 patients were excluded due to unknown race or marital status. Furthermore, our classification of PSA based on the ISUP score may be more applicable than the four-tier division used in the previous SEER manuscript. Therefore, despite using the same database, differences in methodology account for the higher incidence of bone metastases in our study.

Among patients with breast cancer, inflammatory breast cancer was associated with the highest percentage of bone metastases (20.8%), followed by lobular (3.9%) and infiltrating ductal carcinomas (2.9%). In patients with infiltrating ductal carcinoma, the strongest predictors for bone metastases in our study were advanced T stage and N2 or N3. We also found a higher risk for HER2 positive tumors compared to HER2 negative regardless of hormonal status. In a SEER analysis of 229,195 patients with breast cancer diagnosed between 2010 and 2014, 8,295 (3.6%) had bone metastases at presentation (13). Bone metastases were more common in HER2-positive and hormone positive tumors (5.2%) followed by HER2 positive and hormone negative tumors (4.8%), HER2 negative and hormone positive (3.2%) and triple negative (2.8%). Advanced stage and lobular were also associated with an increased risk for bone metastases at presentation in multivariable analysis. In a retrospective German study evaluating 9,625 patients with breast cancer diagnosed between 1992 and 2008, the percentage of bone-only metastases was 18.0% for patients diagnosed between 1992 and 2000 and 28.4% for those diagnosed in 2001 to 2008 (14).

In patients with gastrointestinal tumors, the most common primaries associated with bone metastases were esophageal adenocarcinoma, cholangiocarcinoma, esophageal squamous carcinoma, and gastric adenocarcinoma. In colorectal adenocarcinoma, bone metastases were more common in rectal than colon primaries. While bone metastases were significantly more common in patients with higher T and N stages in univariate analysis, multivariable analysis could not be reliably performed due to the lack of T and N data on more than half of patients with metastatic disease. Bone metastases were rare in the absence of liver or lung metastases. In the OSCER registry, the incidence of bone metastases in patients with colorectal cancer increased from 1.0% in the first year to 2.7% at 10.(4) An earlier study using the SEER database reported an incidence of bone metastases of 1.2%, with risk factors predictive of bone metastases including male gender, higher N stage, rectal site, elevated carcinoembryonic antigen, and lung and liver metastases. (15) However, this study only included patients diagnosed from 2010 to 2015 and used different predictive factors including T stage, co-occurrence of brain metastases and tumor grade. Nevertheless, the three large studies showed a small incidence of bone metastases at presentation in patients with colorectal cancer.

In the National Comprehensive Cancer Network (NCCN) breast cancer guidelines, bone scan or Positron Emission Tomography (PET) are indicated in patients with localized bone pain or elevated alkaline phosphatase but not recommended for the routing use in patients with clinical stage I, II or T3N1. (16) For patients with prostate cancer, the NCCN guidelines recommend bone imaging for patients with unfavorable intermediate, high or very high risk (17).

Untreated bone metastases are associated with skeletal complications at approximately every 3 to 6 months, with an increased frequency during tumor progression (18). Therefore one of the main goals of identifying asymptomatic bone metastases at presentation is to prevent or delay the development of SREs, since once the complications develop, there is often a decline in the quality of life. The use of bisphosphonates have been associated with reduced incidence and delayed onset of SREs in patients with breast cancer, prostate cancer, NSCLC and other solid tumors (19–21). When compared to zoledronic acid, denosumab was associated with delayed time to SRE in breast cancer, prostate cancer and other solid tumors (22–24). More recently, a randomized study showed reduced SREs and hospitalizations in patients with solid treated with prophylactic radiation therapy to high-risk bone metastases (25).

There are several weaknesses in the study including the lack of information on staging methods, genomic profile in lung cancer, and a more comprehensive information on other sites of metastases beyond lymph nodes, bone, brain, lung, and liver, precluding a reliable evaluation of bones as the only site of metastases. Furthermore, it is unknown whether the patients were diagnosed with bone metastases while asymptomatic, due to bone pain or elevated alkaline phosphatase. Nevertheless, our study confirmed the clinically meaningful risk factors for the development of bone metastases in multiple tumors including lung cancer, prostate cancer, and breast cancer. Additionally, our study is unique in that it was dedicated to risk factors for incidence of bone metastases at diagnosis, rather than others that evaluated factors impacting survival in patients with bone metastases. Furthermore, our very large study allowed a detailed evaluation of clinical and pathological risk factors, particularly in prostate and breast cancer.

In summary, our study showed that 5.2% of patients with solid tumors have bone metastases at presentation. Identification of these patients during the initial staging is important since early treatment of asymptomatic lesions is associated with decreased incidence and delay in the development of SREs, with improvement in the quality of life.

## Data availability statement

Publicly available datasets were analyzed in this study. This data can be found here: https://seer.cancer.gov/data/.

## Ethics statement

Ethical approval was not required for the study involving humans in accordance with the local legislation and institutional requirements. Written informed consent to participate in this study was not required from the participants or the participants’ legal guardians/next of kin in accordance with the national legislation and the institutional requirements.

## Author contributions

BK: Conceptualization, Data curation, Investigation, Methodology, Validation, Visualization, Writing – original draft, Writing – review & editing. GC-S: Validation, Visualization, Writing – review & editing. MF: Writing – review & editing. NG: Data curation, Writing – review & editing. FG: Data curation, Formal Analysis, Methodology, Validation, Visualization, Writing – review & editing. PS: Writing – review & editing. RG: Funding acquisition, Supervision, Writing – review & editing. DM: Conceptualization, Data curation, Funding acquisition, Investigation, Methodology, Project administration, Resources, Supervision, Validation, Visualization, Writing – original draft, Writing – review & editing.

## Supplementary material

Supplementary Table 1 Patient demographics.
