# Supplementary material for: Incidence and risk factors for bone metastases at presentation in solid tumors
Source: Front Oncol. 2024 May 10;14:1392667. doi: 10.3389/fonc.2024.1392667 (PMC11116799; doi:10.3389/fonc.2024.1392667)
Supplement: Supplementary Table 1 — Patient demographics. [file Table_1.docx]

**Supplementary Table 1: Patient Demographics**

| **Characteristic** | **Patients with Bone Metastases** | **Total Patients** | **Percentage With Bone Metastases (%)** |
| --- | --- | --- | --- |
| **Gender** |  |  |  |
| Male | 33,247 | 529,442 | 6.28 |
| Female | 22,656 | 545,628 | 4.15 |
| **Age (Years)** |  |  |  |
| 18-65 | 22,812 | 535,754 | 4.26 |
| 66 and older | 33,091 | 539,316 | 6.14 |
| **Race/Ethnicity** |  |  |  |
| White | 43,600 | 860,151 | 5.07 |
| Black | 5,682 | 92,231 | 6.16 |
| Other | 6,471 | 112,033 | 5.78 |
| Unknown | 150 | 10,655 | 1.41 |
| **Brain Metastases** |  |  |  |
| Yes | 7,564 | 20,290 | 37.28 |
| No | 46,215 | 1,051,700 | 4.39 |
| Unknown | 2,124 | 3,080 | 68.96 |
| **Liver Metastases** |  |  |  |
| Yes | 14,492 | 61,210 | 23.68 |
| No | 39,616 | 1,010,746 | 3.92 |
| Unknown | 1,795 | 3,114 | 57.64 |
| **Lung Metastases** |  |  |  |
| Yes | 13,482 | 45,218 | 29.82 |
| No | 39,824 | 1,024,214 | 3.89 |
| Unknown | 2,595 | 5,638 | 46.03 |
